# Tracking of rigid head motion during MRI using an EEG system

**DOI:** 10.1002/mrm.29251

**Published:** 2022-04-25

**Authors:** Malte Laustsen, Mads Andersen, Rong Xue, Kristoffer H. Madsen, Lars G. Hanson

**Affiliations:** ^1^ Section for Magnetic Resonance, DTU Health Tech Technical University of Denmark Kgs. Lyngby Denmark; ^2^ Danish Research Centre for Magnetic Resonance, Centre for Functional and Diagnostic Imaging and Research Copenhagen University Hospital Amager and Hvidovre Copenhagen Denmark; ^3^ Sino‐Danish Centre for Education and Research Aarhus Denmark; ^4^ University of Chinese Academic of Sciences Beijing China; ^5^ Philips Healthcare Copenhagen Denmark; ^6^ Lund University Bioimaging Center Lund University Lund Sweden; ^7^ State Key Laboratory of Brain and Cognitive Science, Beijing MRI Center for Brain Research Institute of Biophysics, Chinese Academy of Sciences Beijing China; ^8^ Beijing Institute for Brain Disorders Beijing China; ^9^ DTU Compute Technical University of Denmark Kgs. Lyngby Denmark

**Keywords:** motion tracking method, prospective motion correction, brain MRI, EEG system, artifact correction

## Abstract

**Purpose:**

To demonstrate a novel method for tracking of head movements during MRI using electroencephalography (EEG) hardware for recording signals induced by native imaging gradients.

**Theory and Methods:**

Gradient switching during simultaneous EEG–fMRI induces distortions in EEG signals, which depend on subject head position and orientation. When EEG electrodes are interconnected with high‐impedance carbon wire loops, the induced voltages are linear combinations of the temporal gradient waveform derivatives. We introduce head tracking based on these signals (CapTrack) involving 3 steps: (1) phantom scanning is used to characterize the target sequence and a fast calibration sequence; (2) a linear relation between changes of induced signals and head pose is established using the calibration sequence; and (3) induced signals recorded during target sequence scanning are used for tracking and retrospective correction of head movement without prolonging the scan time of the target sequence. Performance of CapTrack is compared directly to interleaved navigators.

**Results:**

Head‐pose tracking at 27.5 Hz during echo planar imaging (EPI) was demonstrated with close resemblance to rigid body alignment (mean absolute difference: [0.14 0.38 0.15]‐mm translation, [0.30 0.27 0.22]‐degree rotation). Retrospective correction of 3D gradient‐echo imaging shows an increase of average edge strength of 12%/−0.39% for instructed/uninstructed motion with CapTrack pose estimates, with a tracking interval of 1561 ms and high similarity to interleaved navigator estimates (mean absolute difference: [0.13 0.33 0.12] mm, [0.28 0.15 0.22] degrees).

**Conclusion:**

Motion can be estimated from recordings of gradient switching with little or no sequence modification, optionally in real time at low computational burden and synchronized to image acquisition, using EEG equipment already found at many research institutions.

## 1 INTRODUCTION

Motion is a pertinent problem in brain MR imaging, decreasing image quality and thereby affecting clinical diagnosis and research^1^ and leading to extra costs.^2^ High‐resolution MRI is particularly sensitive to motion, which has triggered investigations into methods of correction.^3^, ^4^

Motion of the brain is mostly rigid body motion and is well characterized by tracking position and orientation (pose) of the imaging volume (FOV). Comprehensively correcting for subject motion requires prospective correction in which motion estimates are used to update the scanner FOV during scanning. This may be needed to avoid artifacts from spin history effects resulting from through‐plane motion in 2D imaging,^5^, ^6^, ^7^ local Nyquist violations in k‐space in multi‐shot sequences,^4^ or inadequate coverage of the brain. However, some MR techniques in which excitation is nonselective and k‐space is sampled in 3 dimensions can be adequately compensated for motion through retrospective correction of samples in k‐space.^8^, ^9^, ^10^, ^11^ This typically relies on pose estimates to infer the actual path traveled in k‐space, used for re‐gridding of k‐space data.^8^, ^12^

Most tracking methods can be categorized as either navigator‐, optical‐, or sensor‐based. Navigators are extra MR measurements interleaved in the target sequence that are registered in either image space^13^, ^14^, ^15^ or k‐space^16^, ^17^, ^18^ to obtain motion estimates without dedicated tracking hardware. Navigators are used widely in measurements when it warrants a prolonged scan time or when they can be acquired during periods of sequence inactivity.^4^ The utility is sequence‐dependent, and navigators may affect sequence timing and the steady‐state magnetization. Interleaved navigators may require extensive sequence modification, and errors can arise when navigators are acquired temporally far from the data being corrected.

With external optical tracking of either facial features^19^, ^20^ or markers fixed to the skin,^21^, ^22^, ^23^ the frequency and accuracy of motion estimation can be excellent, allowing for correction of drift and minor movement. Whereas optical tracking requires minimal sequence modification, there are challenges: calibration of camera and scanner coordinate relation, line‐of‐sight requirements, head coil and bore size limitations, synchronization, and cost of equipment. With markers attached to the skin, there are also challenges associated with placement of the markers, ensuring that it follows skull movement and is not affected by, for example, facial expression.

Another class of tracking methods uses external sensors attached to the subject that track motion by means of the spatial characteristics of the gradient fields. Such methods inherently provide measures in the scanner frame of reference. A commercially available sensor (Robin Medical Inc., Baltimore, MD) based on 3 orthogonally arranged pickups coils with known geometry have been used together with interleaved bipolar gradients.^24^, ^25^ NMR field probes comprising small RF coils enclosing liquid samples have been used with either short interleaved navigator modules (NAVs)^26^, ^27^, ^28^ or sinusoidal kilohertz patterns superimposed on the native gradient waveforms.^29^ Methods combining pickup coils and Hall‐effect magnetometers have shown tracking with minimal pulse sequence modification.^30^ Pickup coils and NMR probes avoid line‐of‐sight limitations and generally offer good coil array and scanner bore‐size compatibility. Attaching sensors, however, may require additional setup time, and although tracking was recently demonstrated using only native imaging gradients,^31^ sequence independent tracking has yet to be achieved, which restricts practical use.^32^, ^33^

The current study introduces CapTrack, a motion‐tracking technique based on the spatial dependence of gradient field changes measured with a modified electroencephalography (EEG) cap. With added carbon wire loops, induced voltages from gradient switching can provide position encoding without extra gradients or tones. This allows for motion tracking without sequence modification and requires no tailored hardware beyond a lightly modified EEG cap and sampling system. For each scan session, the method requires mounting of the cap and a short calibration scan in which the relation between head pose and changes in inductive measures is established. With added carbon wire loops (CWLs), the EEG cap can be deployed quickly without the preparations needed to ensure skin–electrode contact. Motion estimates are acquired in synchrony with the target sequence, with low latency and at flexible temporal resolution. The basic idea and various pilot experiments were introduced in conference abstracts,^33^, ^34^, ^35^, ^36^, ^37^ but the application and performance of the method have not been explored before because a number of fundamental improvements and extensions were needed to demonstrate viability of the method. In this work, per‐slice tracking of multi‐slice echo planar imaging (EPI) is demonstrated, as well as retrospective correction of high‐resolution structural 3D MRI, with direct comparison to volume realignment and interleaved volumetric navigators.

## 2 THEORY

### 2.1 Induced gradient signals in wire loops

For perfectly linear magnetic field gradients, the magnetic field from gradient activity can be described as^38^:

(1) $$\left(\begin{array}{c} B_{x}\left(x,y,z,t\right)\\ B_{y}\left(x,y,z,t\right)\\ B_{z}\left(x,y,z,t\right) \end{array}\right)=\left(\begin{array}{ccc} -\;\frac{1}{2}G_{z}\left(t\right) & 0 & G_{x}\left(t\right)\\ 0 & -\frac{1}{2}G_{z}\left(t\right) & G_{y}\left(t\right)\\ G_{x}\left(t\right) & G_{y}\left(t\right) & G_{z}\left(t\right) \end{array}\right)\left(\begin{array}{c} x\\ y\\ z \end{array}\right),$$

where x,y,z are spatial coordinates with origin at isocenter and the z‐direction along the main magnetic field. $G_{x}\left(t\right),G_{y}\left(t\right),G_{z}\left(t\right)$ are the applied gradient waveforms. The unavoidable concomitant field components $B_{x}\left(x,y,z,t\right)$ and $B_{y}\left(x,y,z,t\right)$ are often neglected in imaging.

From Faraday's law, it follows that the induced signal $v_{i}\left(t\right)$ in wire loop i is a weighted sum of gradient contributions. For a brief period during which motion is negligible,

(2) $$\begin{array}{cc} v_{i}\left(t\right) & =w_{\mathrm{ix}}\frac{\partial {\tilde{G}}_{x}\left(t\right)}{\partial t}+w_{\mathrm{iy}}\frac{\partial {\tilde{G}}_{y}\left(t\right)}{\partial t}+w_{\mathrm{iz}}\frac{\partial {\tilde{G}}_{z}\left(t\right)}{\partial t}+\eta_{i}\left(t\right)\\ & =\dot{\tilde{G}}\left(t\right)w_{i}+\eta_{i}\left(t\right), \end{array}$$

where $\dot{\tilde{G}}\left(t\right)=\left[\frac{\partial {\tilde{G}}_{x}\left(t\right)}{\partial t},\frac{\partial {\tilde{G}}_{y}\left(t\right)}{\partial t},\frac{\partial {\tilde{G}}_{z}\left(t\right)}{\partial t}\right]$ expresses filtered versions of gradient waveform time derivatives resulting from the combined effect of eddy currents, gradient preemphasis, and filters of the EEG system (all assumed linear); and $\eta_{i}\left(t\right)$ is measurement noise. The weights, $w_{i}={\left[w_{\mathrm{ix}},w_{\mathrm{iy}},w_{\mathrm{iz}}\right]}^{T},$ depend on loop geometry, position, and orientation. If the wire loop surrounds a surface $S_{i}$ with infinitesimal area elements ds and local unit normal vector $n\left(x,y,z\right)={\left(n_{x}\left(x,y,z\right),n_{y}\left(x,y,z\right),n_{z}\left(x,y,z\right)\right)}^{T}$, then the weights are given by:

(3) $$w_{\mathrm{ix}}=-\int_{S_{i}}\left[n_{x}\left(x,y,z\right)z+n_{z}\left(x,y,z\right)x\right]\mathrm{ds},$$

(4) $$w_{\mathrm{iy}}=-\int_{S_{i}}\left[n_{y}\left(x,y,z\right)z+n_{z}\left(x,y,z\right)y\right]\mathrm{ds},$$

(5) $$w_{\mathrm{iz}}=-\int_{S_{i}}\left[-\frac{1}{2}n_{x}\left(x,y,z\right)x-\frac{1}{2}n_{y}\left(x,y,z\right)y+n_{z}\left(x,y,z\right)z\right]\mathrm{ds}.$$

**FIGURE 1:**
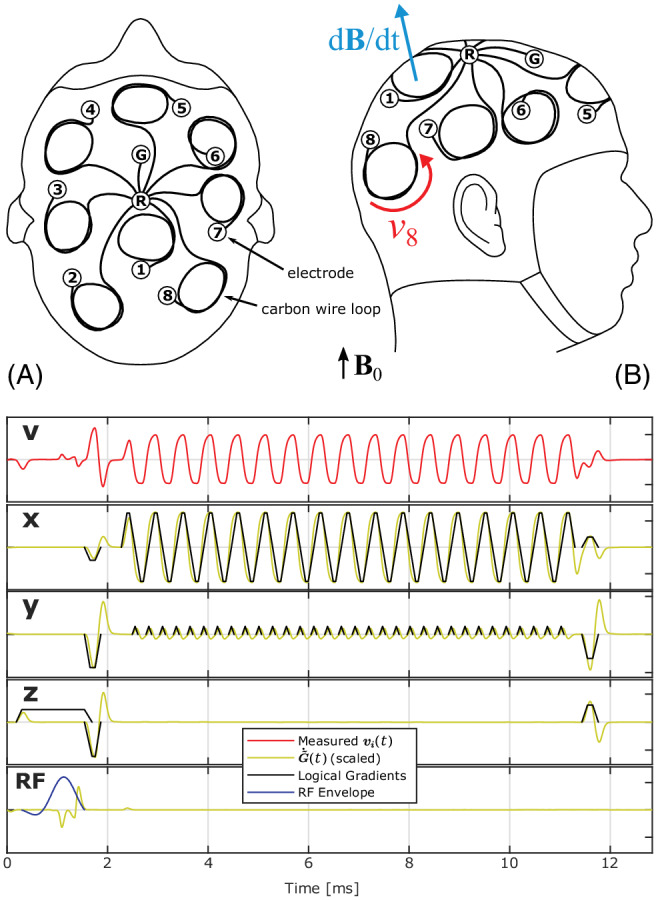
Illustration of the CapTrack setup with the MR‐conditional EEG cap (64‐Channel, Easycap, Herrsching, Germany) seen from above (a) and from the side (b). A subset of 8 electrodes (1 to 8) and the ground electrode (G) were interconnected to the reference electrode (R) using resistive carbon wire. The CWLs were spatially nonoverlapping and distributed approximately uniformly around the EEG cap. Not depicted are wires connecting the electrodes to the EEG preamplifier and electrodes not used in the setup. As indicated, the local derivative of the field may not be directed along the static field due to concomitant gradients. Bottom: The graph depicts a measured signal v(t) for short single‐slice EPI consisting of a weighted sum of columns of $\dot{\tilde{G}}\left(t\right)$ (example channel i). These resemble filtered time derivatives of the pulse sequence and are found by sampling the sequence with only 1 gradient component *x*, *y*, or *z* active, or active RF. Each wire loop recording contains a unique weighted sum dependent on loop position, orientation, and geometry. CWLs, carbon wire loops; EEG, electroencephalography

A recorded signal $v_{i}\left(t\right)$ for a single slice EPI is shown in Figure 1.

### 2.2 Motion‐tracking concept

Equations ((2), (3), (4), (5)) imply that each wire loop i=[1,2,…,I] provides a different mix of filtered gradient waveform derivatives, described by weights $w_{i}$. These 3I vectors can be estimated from any sufficient duration of signal $V\left(t\right)=\left[v_{1}\left(t\right),v_{2}\left(t\right),\ldots ,v_{I}\left(t\right)\right]$ by matrix inversion of Equation (2): The pseudo‐inverse of $\dot{\tilde{G}}\left(t\right)$ multiplied by V(t) (matrices when t is discretized over each such signal “snippet”) minimizes the residual consisting of measurement noise and any unmodelled signal contribution. Changes of weights, ∆w, reflect changes in head pose and therefore can be used for motion tracking. However, without prior detailed knowledge of the sequence and filtering characteristics of EEG and scanner hardware, and with unknown sensor array geometry, it is first necessary to determine the gradient waveforms $\dot{\tilde{G}}\left(t\right)$ and loop orientation dependency of w.

### 2.3 Measuring gradient waveforms

The gradient waveforms are measured with the EEG system prior to performing the “target” scans for which motion correction is needed. This is done using a stationary phantom equipped with the EEG cap and with added CWLs as described in section 3.1. An entire run of the target sequence (all unique phase encoding steps) is repeated for each of the 3 logical gradient waveforms $G_{x}\left(t\right),G_{y}\left(t\right),G_{z}\left(t\right)$ with both the RF transmitter and remaining gradient axes deactivated; thus, all loop channels measure the same waveform but with different amplitudes, according to Equation (2). Waveforms are then determined as the primary singular vector pertaining to the maximum singular value from a singular value decomposition across loop channels. Granted that sequence timings remain unchanged, the gradient waveforms can be reused for all subsequent scans across subjects and orientations.

### 2.4 Calibrating loop orientation dependency

Due to the unknown subject‐dependent geometry of the EEG cap (individual head shape and cap positioning), the loop orientation dependency is determined per subject in a short calibration scan prior to the target sequence. During calibration, a rapid imaging time series is acquired with simultaneous loop voltage recording while the subject deliberately performs free motion, possibly continuous and largely uninstructed. Sufficient motion for each degree of freedom is required to estimate the loop orientation dependency, whereas the exact nature of movement is unimportant. Motion parameter estimates from a rigid body alignment procedure of the calibration images are related to changes in measured gradient waveforms in the loop recordings. For small changes in head position from an arbitrary reference position, small linear changes in weights occur as described by the first‐order Taylor approximation:

(6) ∆w=A⋅∆r+ε,

with ∆w expressing the change of weights from a reference position, and ∆r=[∆x,∆y,∆z,∆θ,∆ϕ,∆ψ] describing relative deviations in head pose with 3 Cartesian translations and 3 Euler angles. The 3I×6 matrix, A, contains partial derivatives of the weights with respect to head pose parameters. The elements of A are found by jointly solving Equation (6) for a number (≥ 6) of calibration positions, that is, minimizing the squared residual error, $\varepsilon^{2}$, in a general linear model.

A rapid 3D‐encoded EPI sequence was used for the calibration scan to keep effects of motion within acquisitions low. The imaging sequence used for calibration need not be similar to the sequence used in the target scan because A and $\dot{\tilde{G}}\left(t\right)$ are independent. Traits of the calibration sequence such as variance and biases from geometric distortions are to some extent inherited. Consideration of alignment biases from geometric distortions caused by, for example, field drift, fat shift, and field inhomogeneity, are therefore important when choosing the sequence for calibration.

### 2.5 Motion tracking during target sequence

After estimating A and $\dot{\tilde{G}}\left(t\right)$, motion parameters are estimated based solely on loop recordings for a new signal snippet V(t) with corresponding weights, w, by matrix inversion of Equation (6). This is an operation with low computational burden that can be performed with low latency either during or after scanning. Due to separation of the tracking signal pathway and the MR signal pathway, the signal snippets V(t) used for calibration and pose estimation can be chosen flexibly to fit image acquisition, and subject pose can be monitored while the sequence is ongoing. For example, motion‐tracking frequency can be adjusted for slice‐wise estimation in EPI, but signal snippets may in general be chosen to include more excitations to ensure adequate gradient switching for reliable tracking.

**FIGURE 2:**
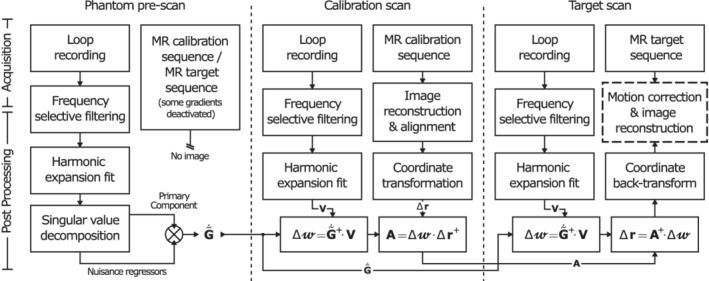
Flow chart of the proposed method. A phantom prescan is used to determine gradient waveforms $\dot{\tilde{G}}\left(t\right)$ by sampling the calibration and target sequence with only 1 gradient channel active for gradient axes *x*, *y*, *z*. A per‐subject calibration scan is used to relate change in weights (∆w) in Equation (2) to changes in subject position (∆r) with calibration matrix A. During the target scan, subject position is estimated from ∆w for each signal snippet V(t). Note that $\dot{\tilde{G}}\left(t\right)$, V(t), ∆w, and ∆r differs between calibration and testing, whereas A remains the same. ⊗ indicates concatenation of nuisance regressors to $\dot{\tilde{G}}\left(t\right)$. ${\left(∼\right)}^{+}$ indicates pseudoinverse. Time dependencies have been omitted for compactness. Additional postprocessing steps are included to reduce bias as explained in section 3.4 and in Supplementary Material A.

A flow chart of the proposed method is provided in Figure 2, with details and additional steps discussed in the following sections.

## 3 METHODS

### 3.1 EEG‐cap setup

For tracking purposes, a subset of 8 electrodes on the MR‐conditional EEG cap (64‐Channel, Easycap, Herrsching, Germany) were interconnected to the reference electrode using resistive wire consisting of insulated bundles of carbon fibers. Terminals of the wire were attached to the electrodes using conductive resin (Electric Paint, Bare Conductive, London, UK). The CWLs were spatially nonoverlapping and distributed randomly around the EEG cap, as illustrated in Figure 1. Because each loop recording provides 3 weights, the 6 rigid body motion parameters can in principle be obtained using 2 loops; however, better accuracy can be achieved using more loops, making the estimation problem overdetermined.

Voltages induced in 8 CWLs were sampled by the EEG sampling system (NeurOne Tesla, Bittium Biosignals Ltd., Kuopio, Finland) with 80 kHz sampling frequency and 16 kHz anti‐aliasing filter. The EEG sampling was synchronized to a 10 MHz clock signal from the scanner, and acquisition was triggered by the scanner at sequence start. Scanner clock synchronization ensures waveform consistency between signal snippets without interpolation, which may be compromised by high‐frequency signals picked up after filtering in electronic signal pathways of the EEG equipment.

### 3.2 Safety aspects

The employed EEG cap has 10 kΩ safety resistors at each electrode terminal and is approved for use at 3 tesla. Using conductive materials in an MR scanner, especially when in contact with the subject, requires extra safety considerations due to induced currents and accompanying electric fields.

Three measures were taken to eliminate risks of RF burns: (1) The cap electrodes used for motion tracking and the leads added for their interconnection were insulated from the patient; (2) the >100 Ω impedances of the interconnecting loops were chosen high relative to those of similar‐sized loops simulated and verified for another study to give insignificant heating^39^; additionally, the 10 kΩ safety resistors at electrode lead terminals ensure that impedances are within the normal range of EEG‐MRI for which the equipment is designed; and (3) using the conductive resin, the resistance was distributed around the loops, which is an established way to prevent high local E‐fields.

The RF coupling was verified to be insignificant at relevant frequencies using a network analyzer and decoupled dual pickup coils, both on individual leads and using the complete setup, unmounted and mounted to the subject. RF coupling was verified to cause negligible intensity distortions and other artifacts near CWLs in a B1‐sensitive variable tip angle rapid acquisition with relaxation enhancement sequence with the cap mounted on a CSF‐like phantom.

### 3.3 Frequency‐selective filtering

Improved SNR was achieved by reducing η(t) in Equation (2) by filtering the loop recordings with a 100 Hz high‐pass filter to remove DC and low‐frequency transient changes, for example, electromotive forces from ballistocardiac effects or loop motion in the strong static field, which are not the focus of this study but can alternatively be used for tracking.^32^

Similarly, gradient switching causes mechanical vibration of the gradient coils, which may propagate into the subject or leads, causing induced signal that is not described by gradient contributions. The frequencies depend on sequence timing as well as vibrational modes of the hardware and can be colinear with gradient waveforms.

**FIGURE 3:**
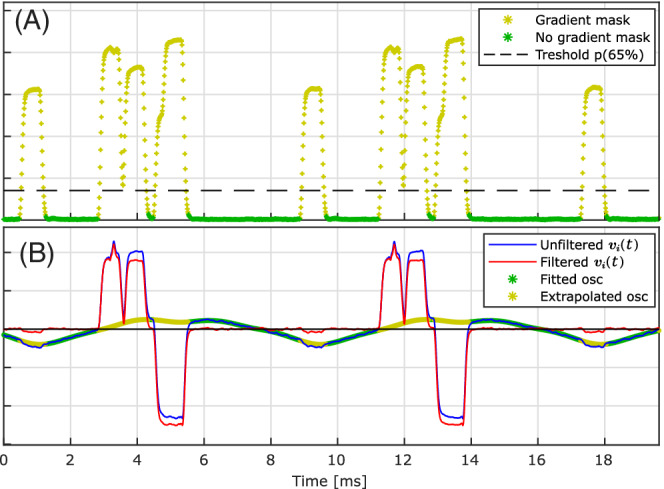
Fitting mechanical vibration for a 3D‐GRE sequence with a low‐frequency harmonic expansion [120–400 Hz]. (a): Periods of gradient ramping are determined by averaging $\left|\dot{\tilde{G}}\left(t\right)\right|$ over TR intervals and thresholding at the 65‐percentile followed by binary dilation with a kernel of ∼0.1 ms length. (b): The gradient mask is used to fit a harmonic expansion to periods free of gradient ramping (green). The resulting fit of the oscillations is evaluated at remaining indices (yellow) and subtracted from electrode voltages $v_{i}(t)$ (blue) to filter the input signal. osc, oscillation

To reject signal components from vibration, periods with gradient ramping are determined by averaging $\left|\dot{\tilde{G}}\left(t\right)\right|$ across TR intervals and thresholding at the 65 percentile (found appropriate for the gradient duty cycle in the target sequence) followed by binary dilation (Figure 3A). Mechanical vibrations are approximated by fitting a low‐frequency harmonic expansion ([120–400 Hz] in accordance with a previous study using the same scanner^40^) to periods free of gradient ramping and removed from V(t) by subtraction (Figure 3B). This was found to suppress mechanical resonances without significantly affecting gradient induced signal. Additionally, the leads connecting the cap and the preamplifier were placed on foam pads to reduce mechanical coupling to the scanner bore.

### 3.4 Stability and bias minimization

Improved accuracy of motion estimates was achieved by adding measures to reduce bias. Nuisance regressors are introduced to avoid structured residuals and correspondingly biased estimates. They are included in $\dot{\tilde{G}}\left(t\right)$ in Equation (2) whereas their corresponding weights (w) are omitted in Equation (6).

The gradient derivatives themselves are typically partially collinear because gradients are often switched in synchrony. Even when switching is asynchronous, subsequent vibration, eddy currents, and cross talk between loop channels may introduce collinearity that is not directly related to gradient switching but can bias motion parameter estimates if not modeled.

Nuisance regressors are calculated as singular vectors pertaining to the nonleading singular values across conductive loop channels in the phantom prescan. The number of regressors to include is determined from the “elbow” of the ordered singular values (scree test).^41^ In this study, 2 nuisance regressors per gradient axis were chosen. Additional nuisance regressors are included in Equation (2) by sampling an RF pulse signature by turning off all gradients, as seen in Figure 1(RF). Nuisance regressors are particularly important for minimizing the residual signal when gradient activity within a signal snippet V(t) is near 0 for any gradient channel. An example is phase encoding in 3D sequences, where gradient activity in the outer phase‐encoding loop is lowest when reading out the central slices of k‐space.

Additionally, robustness of the calibration was improved by a coordinate transformation of motion parameters from rigid body alignment that orthogonalizes the principal modes of movement. This is described in further detail in Supplementary Material A. Consequently, the length of the calibration period could be decreased.

### 3.5 Retrospective motion correction

Motion estimates derived from the inductive recordings are applied retrospectively to correct k‐space samples using the RetroMoCo‐Box^8^ based on the Michigan Image Reconstruction Toolbox.^12^ They are interpolated in time to yield 1 unique motion estimate per line of k‐space using piecewise cubic Hermite interpolating splines enforcing smooth evolution. Each line of k‐space is transformed and re‐gridded individually.

### 3.6 Motion experiments

Efficacy of CapTrack in terms of motion estimator accuracy, precision, and frequency was assessed for 2 scenarios: (1) high‐resolution 3D structural T_1_‐weighted gradient echo imaging (T_1_w 3D‐GRE), and (2) dynamic multi‐slice EPI as used in fMRI (2D‐EPI). High‐resolution imaging is a relevant use case suited for indirectly evaluating the accuracy of motion tracking due to high susceptibility to motion, whereas multi‐slice EPI can benefit from low‐latency tracking “per‐slice” over typical “per‐volume” image‐based tracking, especially with prospective acquisition updating that prevents spin history effects.^42^, ^43^ The retrospective correction allowed for direct comparison to interleaved navigator tracking and correction.

Images were acquired using a 3 tesla MRI system with a 32‐channel head coil (Achieva, Philips Healthcare, Best, The Netherlands).

A T_1_‐weighted 3D spoiled gradient‐echo (T_1_w 3D‐GRE) structural sequence was used as the target for motion correction. The flip angle was chosen for good gray/white matter contrast. The inner/outer phase‐encoding ordering was linear/center‐out, with head−feet readout direction chosen to avoid fold‐in artifacts.

For comparison of tracking efficacy, the T_1_w 3D‐GRE sequence was interleaved with EPI volumetric NAVs^14^ at each outer phase‐encoding step using an interleaved scanning framework available on Philips Healthcare MRI scanners for research use.^44^, ^45^ The navigator flip angle was set low to minimize the impact on the target sequence. Field drift was seen to cause parameter drift (especially in *y*‐translation); therefore, each NAV was accompanied by a short readout for dynamic excitation frequency updating, which corrected for drift but slightly increased random noise in *y*‐translation parameters.

A 3‐mm isotropic multi‐slice EPI time series (2D‐EPI) was used to demonstrate per‐slice tracking. A TR of 4.5 s was chosen to leave 2.1 s of sequence inactivity for motion between volume acquisitions for comparison to rigid body alignment.

Parameters for all sequences used are listed in Table 1. The volume repetition times of the sequences were chosen as multiples of the EEG sample intervals (0.0125 ms) to ensure consistently measured gradient waveforms between repetitions.

**TABLE 1:** Sequence parameters for the 4 sequences. Listed are parameters for the fMRI‐like multi‐slice sequence (2D‐EPI), the structural T_1_w 3D spoiled GRE, the 3D EPI‐based NAVs, and the 3D non‐EPI–based navigator modules (FFE‐NAVs).

Sequence	TE/TR [ms]	Dynamic Time [s]	Total Scan Time [s]	Flip Angle [deg]	Voxel Size [mm]	Matrix Size
fMRI (2D‐EPI)	17.9/36.3	2.4/4.5	225	80	3 × 3 × 3	80 × 80 (65 slices)
T_1_w 3D‐GRE	3.0/6.5	1.6	420	14	1 × 1 × 1	240 × 240 × 240
NAVs (3D EPI)	5.8/14.0	0.46	111	2.0	7.1 × 8.2 × 7.0	36 × 31 × 29
FFE‐NAVs	0.64/1.3	1.0/3.0	240	1.7	7.1 × 6.9 × 7.0	36 × 37 × 29

Abbreviations: FFE, fast field echo; GRE, gradient echo; NAV, navigator.

TR is here the time between excitations, which may differ from the conventional definition of TR indicating time between excitations of the same volume. Dynamic time is time‐per‐volume acquisition (without/with interleaved breaks for 2D‐EPI and FFE‐NAVs), except for T1w 3D‐GRE, where it is time per outer phase‐encoding step. Total scan time is time spent per sequence during 1 full recording of the target scan (without navigators for T1w 3D‐GRE).

For T_1_w 3D‐GRE scans, the CapTrack motion‐tracking frequency was chosen to match that of the navigator, yielding 1 position estimate for each of the 240 acquired planes in k‐space. The snippet length was chosen as 2 TRs (3122 ms) so that adjacent snippets had 50% overlap. Loop recording during navigator periods was not used for calibration or for motion estimation with CapTrack to make comparison between navigators and CapTrack fair. For the multi‐slice EPI sequence, the motion‐tracking frequency was chosen to yield estimates for each of the 65 acquired slices, resulting in 27.5 Hz tracking frequency.

#### 3.6.1 In vivo experiments

In vivo experiments were conducted with 3 healthy adult volunteers wearing the interconnected EEG cap. Because the study did not involve medical research as defined in national Danish regulations but only addressed data quality, ethical permission is not needed and cannot be obtained. Only senior researchers involved in the study participated as subjects, and only after informed consent.

An 80 seconds calibration scan with continuous dynamic NAV scanning without interleaved breaks was acquired during deliberate free motion prior to the structural scan. Subjects were asked to conduct continuous head motion during the calibration, covering roughly the modes of motion possible in the restricting head coil. The exact nature of movement was otherwise uninstructed. The sequence used for calibration was identical to the interleaved navigator, without any additional interleaved gradients or tones. Motion parameters from rigid body alignment (using Statistical Parametric Mapping version 12, SPM12, Wellcome Centre UCL, London, UK) of the calibration images were used for CapTrack calibration.

Five T_1_w 3D‐GRE images (4 with NAVs) were acquired for each subject. These were instructed to lie still during 2 scans and to perform deliberate motion as described above during 3 scans. Subjects received feedback on the amount of motion corruption between scans.

A 50‐volume dynamic EPI‐2D scan was acquired for all subjects, who were instructed to perform interscan motion during the first 30 volumes and intrascan motion for the last 20 volumes.

#### 3.6.2 Phantom experiments

Phantom experiments were performed using a spherical agar phantom with embedded plastic tubing to provide adequate structure for rigid body alignment using SPM12. The EEG cap was placed over the spherical phantom, which was mounted on a 2 m wooden handle that enabled an operator to manually move it during scanning while avoiding field disturbance near the imaging volume. For evaluating the tracking performance, 3 structural scans were acquired with variable amounts of motion, and 2 structural scans were acquired with the phantom stationary at 2 different positions. For the phantom study, the EPI‐based navigators exhibited severe geometric distortions due to shim imperfections; thus, 3D (non‐EPI) fast field echo navigator modules (FFE‐NAVs) with 1 s dynamic scan time were used as ground truth. Dynamic scanning of FFE‐NAVs interleaved with 2 s breaks were used for calibration during operator‐induced step‐wise interscan motion, which resulted in a long calibration time of 180 s. These long durations are not necessary for in vivo scanning, only for validation of principles and implementation.

#### 3.6.3 Performance metrics

The CapTrack accuracy was established by comparison to rigid alignment (using SPM12) of navigators and volumes in the EPI time series, which was taken as ground truth.

Changes in average edge strength (AES) (standard measure of image sharpness) of the structural scans were compared after using either tracking method for retrospective correction. Prior to AES calculation, corrected, uncorrected, and reference images were realigned to a common frame with rigid realignment (SPM12) using sinc interpolation, and image dimensions were cropped tightly around the brain to avoid bias from regions of nonrigid movement (e.g., neck or ears). Cropping was guided by a brain mask extracted from the no‐motion image using FSL brain extraction tool (FSL‐BET) (FMRIB, Oxford, UK) with the “robust” option. AES was calculated and averaged over sagittal, coronal, and axial directions using the AES toolbox.^46^

## 4 RESULTS

For the multi‐slice EPI sequence, CapTrack resulted in “per‐slice” motion estimation, with an update rate 65 times more frequent than the typical per‐volume rigid body alignment. For high‐resolution 3D structural T_1_ weighted imaging, both CapTrack and NAVs achieved the same total of 240 estimates. CapTrack tracking did not increase scan time during the target sequence, but the scan time spent on calibrating the system prior to scanning was 80 s for the whole session compared to 111 s additional scan time spent per structural scan on NAVs.

**FIGURE 4:**
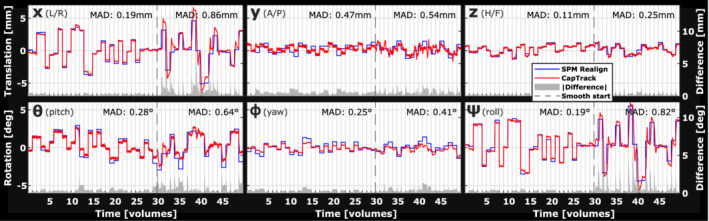
Tracking during a dynamic series of fMRI‐like 2D‐EPI (subject 3). The subject was instructed to move only during interleaved breaks in the first 30 volumes (interscan motion), and to perform continuous smooth motion for the remaining 20 volumes (intrascan motion). MAD between navigator (blue) and CapTrack (red) motion parameters is increased during intrascan motion due to CapTrack parameters more closely resembling the underlying smooth motion. MAD, mean absolute difference

Comparing tracking curves for 2D‐EPI in Figure 4 reveals close resemblance between SPM12 alignment and CapTrack that, however, provides smoother tracking curves during continuous motion. Table 2 lists mean absolute difference between the 2 methods for periods with interscan and intrascan motion separately. Average mean absolute difference was [0.14 0.38 0.15] mm, [0.30 0.27 0.22] degrees for interscan motion, which increased to [0.66 0.48 0.27] mm, [0.54 0.70 0.60] degrees during intrascan motion. Applying motion estimates from CapTrack and per‐volume rigid body alignment both resulted in a substantial increase of the temporal SNR after retrospective volume alignment (Supporting Information Figure S1).

**TABLE 2:** MAD between rigid alignment of EPI volumes and CapTrack tracking parameters for periods of interscan motion, and periods of intrascan motion in a 2D‐EPI time series. Parameters differ more during intrascan motion mainly due to the higher temporal resolution of CapTrack

	Translation [mm]			Rotation [deg]		
MAD: 2D‐EPI	*x*	*y*	*z*	*θ*	*φ*	*ψ*
Subject 1						
Interscan	0.16	0.50	0.22	0.40	0.37	0.33
Intrascan	1.00	0.70	0.45	0.76	1.50	0.79
Subject 2						
Interscan	0.065	0.17	0.11	0.20	0.19	0.15
Intrascan	0.12	0.19	0.11	0.20	0.13	0.18
Subject 3						
Interscan	0.19	0.47	0.11	0.28	0.25	0.19
Intrascan	0.86	0.54	0.25	0.64	0.41	0.82
Mean						
Interscan	0.14	0.38	0.15	0.30	0.27	0.22
Intrascan	0.66	0.48	0.27	0.54	0.70	0.60

Abbreviation: MAD, mean absolute difference.

**FIGURE 5:**
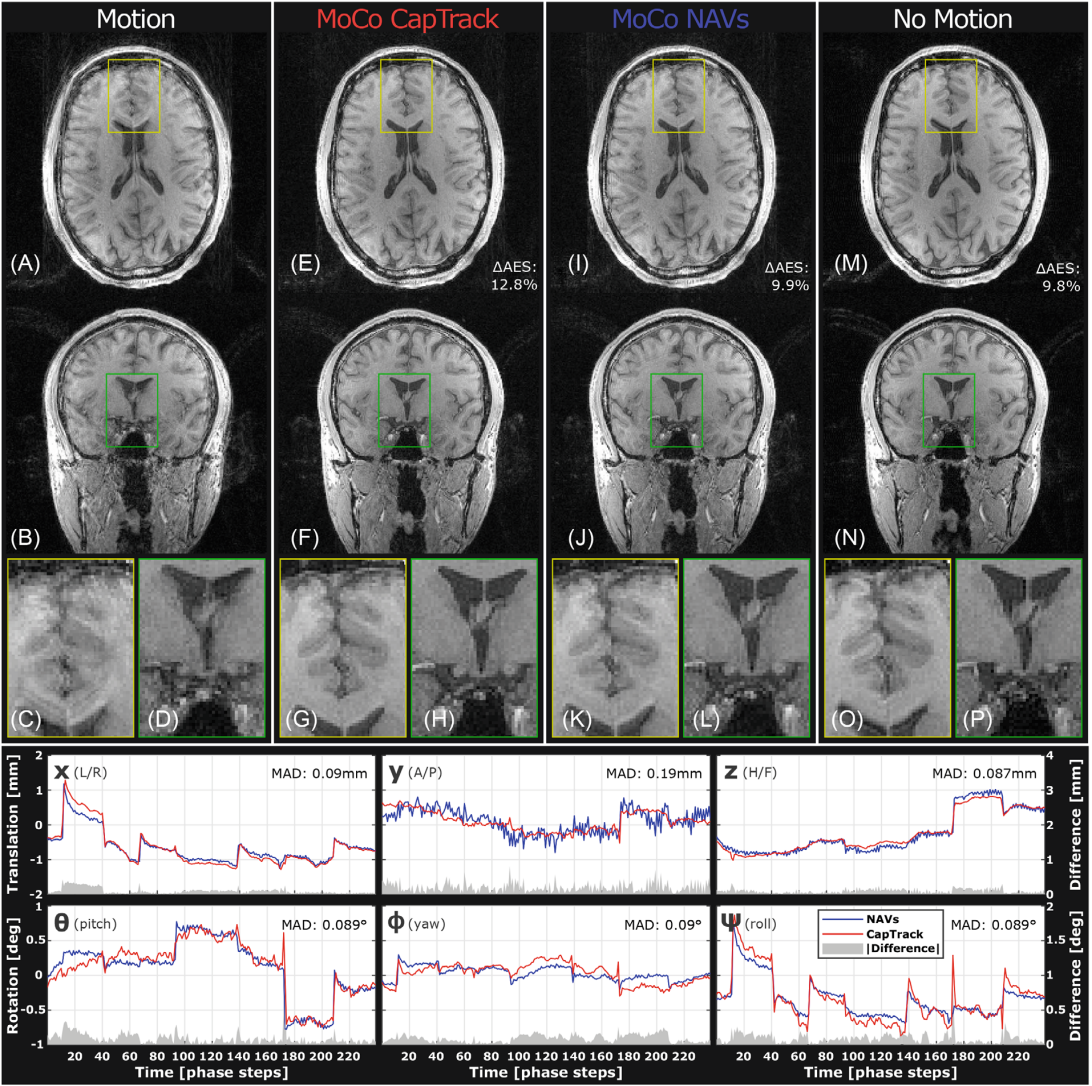
Retrospective motion correction of a structural scan with moderate deliberate motion (Table 4: subject 2, trial 4). Maximum absolute displacements of 1–2 mm (*x*,*y*,*z*) and 0.5–1.5 degree (θ,ϕ,ψ) are present in motion parameters from either tracking method. Tracking curves from CapTrack (red) and NAVs (blue) show high degree of similarity (gray) (low MAD: [0.09 0.19 0.087] mm, [0.089 0.09 0.089] degrees) with largest discrepancy in *y*‐translation. Visual sharpness is substantially improved after retrospective correction using either tracking method (e‐l). Both methods lead to an AES increase of 12.8/9.9% for CapTrack/NAVs respectively. AES, average edge strength; MAD, mean absolute difference

Figure 5 depicts an example of tracking and retrospective correction of moderate head motion during T_1_w 3D‐GRE. Clear improvements in visual sharpness and AES are observable when applying retrospective corrections using CapTrack. In Figure 5, only slight deviations between navigator and CapTrack motion parameter estimates are seen, although navigator *y*‐translation appears more noisy and CapTrack exhibits more spiking during sudden motion. Table 3 lists average mean absolute difference between CapTrack and NAV estimates.

**TABLE 3:** MAD between rigid alignment and CapTrack tracking parameters averaged over all trials for in vivo fMRI‐like multi‐slice 2D‐EPI images with interscan motion (fMRI 2D‐EPI), in vivo structural T_1_W‐3D GRE with interleaved 3D‐EPI navigators (NAVs), and phantom structural T_1_W‐3D GRE with interleaved FFE‐based 3D navigators (FFE‐NAVs), respectively. Close similarity to rigid alignment is observed across the 3 tested scenarios

	Translation [mm]			Rotation [deg]		
MAD: Total	*x*	*y*	*z*	*θ*	*φ*	*ψ*
fMRI 2D‐EPI	0.14	0.38	0.15	0.30	0.27	0.22
NAVs (3d‐EPI)	0.13	0.33	0.12	0.28	0.15	0.22
FFE‐NAVs	0.13	0.22	0.22	0.09	0.19	0.16

Abbreviation: MAD, mean absolute difference; FFE, fast field echo; GRE, gradient echo; NAV, navigator.

**FIGURE 6:**
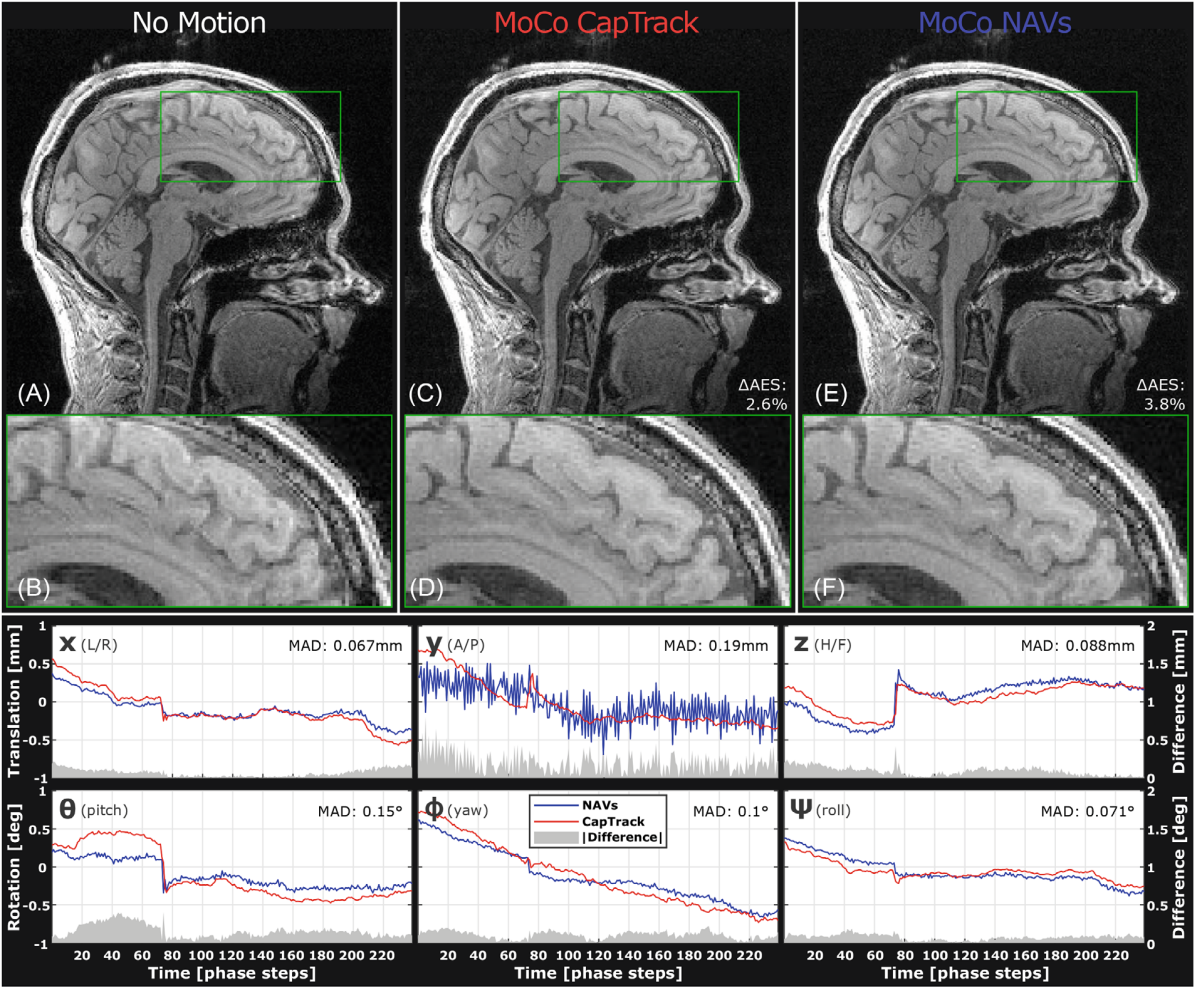
Retrospective motion correction of T_1_w 3D‐GRE in which the subject is instructed to lie still (Table 1: subject 1, trial 2). Involuntary motion of up to 1 mm and 1 degree is visible with both tracking methods, and tracking curves show a high degree of similarity. The highest discrepancy is seen in *y*‐translation where NAVs (blue) seem noisier than CapTrack (red). A discernible increase in visual sharpness is evident after retrospective correction (c‐f) using either tracking method, especially in frontal area (d,f), and both methods lead to a slight increase to AES of 2.6/3.8% for CapTrack/NAVs, respectively. GRE, gradient echo

Figure 6 displays tracking of involuntary motion of a subject attempting to lie still. Tracking curves reveal minor unintentional movement. After retrospective correction, an appreciable improvement in visual sharpness and a small increase in AES of 2.6%/3.8% are observed for CapTrack and NAVs, respectively.

Table 4 lists change to AES after retrospective correction of each trial. Average improvements to AES in in vivo trials in volumes with instructed movement were 12%/13% for CapTrack/NAVs, respectively, and −0.39%/1.8% in volumes for which the subject was instructed to lie still.

**TABLE 4:** AES before and after retrospective correction of all T_1_w 3D‐GRE using motion estimates from either 3D FFE‐NAVs, 3D‐EPI NAVs, or CapTrack. Entries marked with (*) are potentially affected by stripe artifacts arising from unwanted coherence from the interleaved navigator

	Laying Still		Instructed Movement		
Average Edge Strength	Trial 1	Trial 2	Trial 3	Trial 4	Trial 5
Phantom					
Uncorrected	365(*)	245	152(*)	133	148
MoCo FFE‐NAVs	367(+0.4%)(*)	—	210(+38.3%)(*)	—	—
MoCo CapTrack	369(+0.9%)(*)	238(−2.8%)	278(+82.8%)(*)	203(+52.7%)	188(+27.9%)
In vivo subject 1					
Uncorrected	264	254	170	174	190
MoCo NAVs	266(+0.7%)	263(+3.8%)	211(+24.0%)	203(+16.4%)	—
MoCo CapTrack	255(−3.5%)	260(+2.6%)	206(+21.2%)	197(+13.0%)	204(+7.0%)
In vivo subject 2					
Uncorrected	249	256	291	223	223
MoCo NAVs	264(+6.1%)	263(+2.8%)	228(+13.7%)	245(+9.9%)	—
MoCo CapTrack	270(+8.6%)	254(−0.6%)	232(+15.5%)	251(+12.8%)	256(+14.6%)
In vivo subject 3					
Uncorrected	238	239	175	181	156
MoCo NAVs	241(+1.2%)	230(−3.6%)	193(+10.3%)	193(+7.0%)	—
MoCo CapTrack	227(−4.5%)	227(−5.0%)	187(+6.9%)	189(+4.4%)	170(+9.2%)

Abbreviation: AES, average edge strength; MoCo, motion correction; FFE, fast field echo; GRE, gradient echo; NAV, navigator.

Long relaxation times of the phantom (T_1_/T_2_ ≈ 3000/170 ms) lead to subtle interference between the target sequence and the FFE‐NAVs that affected navigator precision negatively and lead to faint stripe artifacts visible in Supporting Information Figure S2. CapTrack thus outperformed FFE‐NAVs in phantom experiments, reaching 54.2% average improvement to AES in phantom trials with movement. Applying CapTrack correction to stationary phantom scans did not decrease AES except insignificantly and inconsistently as a result of random noise, nor did it result in any observable blurring. The SD of CapTrack motion estimates for the 2 stationary phantom scans was [0.084 0.081 0.038] mm, [0.024 0.034 0.038] degrees.

## 5 DISCUSSION

Results are indicative of the viability of CapTrack as a motion‐tracking method using an EEG setup with added CWLs, based on a calibration scan, without the need for sequence modification. Pose estimates can be achieved for any period with sufficient gradient switching. The inhomogeneous gradient fields involving concomitant field components potentially allow for adequate position encoding with a single switching gradient field, making the approach applicable for most sequences. We have demonstrated fast and accurate tracking in 2 commonly used sequences: (1) high‐resolution 3D structural T_1_‐weighted images (T_1_w 3D‐GRE), and (2) dynamic multislice EPI as used in fMRI (2D‐EPI).

CapTrack resulted in both perceived and measured improvements to image sharpness (AES) in all in vivo trials with deliberate motion during T_1_w 3D‐GRE (Table 4). Improvements to AES and perceived sharpness were observed across a wide range from minor involuntary motion to severe intentional motion. The degree of improvement in vivo was comparable but slightly inferior to retrospective correction based on interleaved NAVs as expressed by AES after motion compensation (Table 4). In phantom experiments, CapTrack vastly outperformed navigators in terms of improvement to AES, presumably in part due to unwanted interference between navigators and target sequences resulting from long coherence times (Supporting Information Figure S2). AES is also a questionable quality measure for phantom experiments because the additional sharp edges introduced by ghosting and ringing could affect the Canny edge detection. However, despite these limitations the phantom experiments clearly validate the CapTrack implementation. Tracking curves from NAVs and CapTrack show close resemblance (Figures 5 and 6) deviating on average by [0.13 0.33 0.12] mm, [0.28 0.15 0.22] degrees (Table 3).

The flexible motion‐tracking frequency of the approach was demonstrated with per‐slice estimation in 2D‐EPI and motion estimation per k‐space plane in structural T_1_w 3D‐GRE. In 2D‐EPI, CapTrack provided motion estimates for every slice acquisition, 65 times more frequent than standard rigid body alignment. In periods of interscan motion, CapTrack motion estimates closely resemble rigid volume realignment (mean absolute difference: [0.14 0.38 0.15] mm, [0.30 0.27 0.22] degrees), whereas they differ during intrascan motion in which CapTrack shows expected smooth motion (Figure 4) (Table 2). Differences during periods of interscan motion are partly due to accidental intrascan motion reflected in CapTrack estimates. We chose not to report residual pixel errors after realignment because these are biased by geometric distortions and intrascan motion. CapTrack motion estimates led to increased temporal SNR after retrospective volume alignment of 2D‐EPI (Supporting Information Figure S1). The largest temporal SNR improvement was seen when using motion estimates from rigid body alignment (SPM12), which is expected as the algorithm minimizes the temporal SD of pixel values. Better performance correction based on CapTrack is expected when the increase in motion‐tracking frequency is utilized for prospective updating to compensate through‐plane motion^5^, ^6^, ^7^, ^42^, ^43^ or when using tracking curves as nuisance regressors in fMRI analysis.^5^, ^47^ The tracking accuracy may change when gradients are updated prospectively, however, and the performance demonstrated here is therefore not necessarily indicative of that attainable. Notably, with only 10 input channels (8 loop channels, reference channel, and ground) and limited need for common‐mode rejection, significantly simpler hardware than a full EEG system may alternatively be used for signal sampling.^40^

For 3D‐GRE, center‐out acquisition was chosen for the outer loop, without impacting image contrast, so that motion estimation could be based on signal from 2 consecutive k‐space planes acquired with phase‐encoding gradients of opposite polarity. This scheme was chosen so that the angle between columns of $\dot{\tilde{G}}\left(t\right)$ would remain mostly similar for each signal snippet V(t). Collinear/multicollinear predictors is a general concern in linear estimation when considering the contribution of individual predictors to a model. Estimated weights w therefore only reflect the true importance of each predictor in a setting with near constant collinearity between columns of $\dot{\tilde{G}}\left(t\right)$, which is compromised for sequences with high degree of dissimilarity in gradient switching between repetitions. This limitation can potentially be mitigated with the addition of L2 regularization (ridge regression) in Equation (2) designed to keep estimated parameters stationary during no motion, further contributing to making the approach sequence independent. Such regularization, and the inclusion of a weak Kalman filter, could potentially also allow for shorter “within‐TR” tracking intervals.

CapTrack involves CWLs isolated from the subject and therefore requires the patient to wear a cap that can be mounted in seconds. Preliminary testing has shown that the method also works for normal EEG‐fMRI in which the scanned subject forms part of the circuits instead^36^ (the EEG signal components were sufficiently small compared to gradient‐induced signals). Interconnecting electrodes for motion tracking circumvents the complexity of connecting electrodes to the scalp and the challenges with drifting impedances caused by sweating, drying electrode gel, or loose contacts. For EEG‐fMRI, some EEG input channels could advantageously be assigned to CWLs aimed at motion tracking. Simultaneous EEG recording could additionally benefit from denoising capabilities enabled by the “clean” gradient artifact recording from the CWLs,^48^, ^49^ from the basis set in $\dot{\tilde{G}}\left(t\right)$ to effectively describe and remove the position dependent gradient artifact,^50^, ^51^, ^52^ and from the recorded motion parameters to filter out the induced current from moving in the static field.^48^, ^49^, ^51^, ^52^ In the current study, an MR‐conditional but adapted EEG cap was chosen to demonstrate that a minimally changed standard EEG‐MRI setup is suited for tracking, but a dedicated smooth cap with integrated high‐impedance wire loops could improve patient comfort and prevent minor displacement of wires relative to the cap, which is a likely source of inaccuracies. EEG caps with integrated CWLs for the purpose of MR‐artifact rejection in EEG^48^, ^49^ have recently been announced as a commercial product and would be interesting to test with CapTrack.

CapTrack is dependent on an approximately linear relationship between changes in the induced signal and in subject position. Whereas rigid‐body motion of the loops is not a necessity, irreversible lead motion and deformation can lead to estimator drift. EEG caps are designed to minimize electrode displacement during light movement, like varying facial expression, and although it is possible to move the cap somewhat independently of the head, a fixed relation between the EEG cap and the subject seems well preserved for the motion possible in a head coil despite the fact that it is difficult to quantify to what extend cap deformation contributes to tracking noise. Tracking discrepancy curves from previous work^37^ indicate that CapTrack tracking noise is largely independent of distance from the starting position or isocentre of the scanner. In other work, an EEG cap was found sufficiently robust to measure k‐space trajectory inaccuracies.^53^

The bias minimization used in CapTrack facilitated significant improvements to motion estimator accuracy and frequency over previous work with gradient field‐based motion tracking using coils with unknown geometry,^33^, ^34^, ^35^, ^36^, ^37^ bringing tracking accuracy in line with systems using orthogonally arranged coils^24^, ^25^, ^30^ while eliminating the need for additional interleaved or superimposed gradients.^24^, ^25^, ^30^, ^33^ Compared to tracking head velocity based on transient electromotive forces signals from motion in the static field,^32^ using gradient‐induced signals conveniently provides estimates in the scanner frame of reference and offers much better SNR. Comparison to other external tracking systems is possible to the extent that they have been compared to interleaved navigators with parameters similar to those used in this study. CapTrack is unlikely to rival the tracking precision of current optical tracking methods, for example.

Using the proposed method with confounding head coils that prevent line of sight or mounting of large probes is a main use case. CapTrack is especially attractive when interleaving NAVs or superimposing tones would compromise the performance of the target sequence. Here, 80 s were spent on calibrating the system to each subject compared to an increase in scan time of 115 s per structural scan with interleaved navigators. The need for per‐subject calibration is a drawback, but this is somewhat comparable to cross‐calibration needed for optical tracking methods with variable patient–camera relation,^4^ which is often performed per subject. Compared to optical tracking, the calibration scan for CapTrack requires more compliance from the subject, which can potentially be mitigated with passive motion by dynamically inflatable pillows integrated in the head coil or cap, for example.

The demonstrated retrospective correction improves the image quality in motion‐corrupted scans without the tracking uncertainty compromising uncorrupted scans as when scanning a still phantom. However, some artifacts remain due to limitations of retrospective correction, especially in scans with abrupt motion exceeding 5 mm/degree. Artifacts primarily arise from Nyquist violations locally in k‐space, limited motion‐tracking frequency, uncompensated field fluctuations, and lack of sampling density compensation. For large sudden rotations, the lack of density compensation and undersampled regions of k‐space introduce ringing artifacts that counteract the benefits of correction, which is a limitation of the retrospective motion correction and reconstruction rather than the motion tracking itself.^4^, ^54^ These can be avoided when prospectively updating the scanner FOV and with reacquisition of corrupted k‐space lines. Simulation of the best attainable retrospective correction using the measured motion to corrupt images in silico followed by correction could serve as a comparison to “best‐case correction” but was beyond the scope of the study.^54^

The low computational complexity and the time‐synchronized nature of CapTrack lends itself well to a prospective implementation, as demonstrated earlier,^35^ requiring only online interfacing to the scanner and the multiplication of a transformation matrix with the 3 first columns of $\dot{\tilde{G}}\left(t\right)$ in Equation (2) whenever the scanner FOV is updated. However, prospective updating is practically demanding in terms of scanner control and is beyond the scope of this study. In addition to reconstruction issues, remaining motion artifacts arise from the geometric distortion of EPI during calibration, motion during signal snippet acquisition, violation of the linearity requirement, and calibration measurement not necessarily representing all relevant motional degrees of freedom around the reference position.

Finally, the limit of CapTrack precision depends mainly on the gradient activity and its temporal and directional diversity. Field strength and imaging acceleration are only of limited importance via their effects on the choice of calibration data acquisition and the resulting image quality. Improvements to calibration and regularization terms, prospective updating, and a cap designed for the purpose of tracking represent potential further advancements.

## 6 CONCLUSION

CapTrack is a novel approach to real‐time tracking of head motion relying on gradient‐induced signals in CWLs recorded using a minimally adapted EEG cap and EEG equipment already found at sites doing simultaneous EEG‐fMRI. The method was demonstrated in experiments involving both a phantom and healthy volunteers, and substantial improvements in image quality were observed in all settings. Although this study is far from exhaustive in terms of elucidating the general performance, the method is shown to offer precision comparable to that of the applied 460 ms 3D navigator, allowing for correction of millimeter‐/degree‐sized unintentional motion in healthy volunteers under relevant conditions, in particular multi‐slice EPI and 3D structural imaging with nonselective RF pulses. The method is applicable to most commonly used sequences without sequence modification.

## CONFLICT OF INTEREST

Malte Laustsen is now an employee of TracInnovations, Ballerup, Denmark. Mads Andersen is an employee of Philips Healthcare, Copenhagen, Denmark.

## Supporting information


**Figure S1.** Map of temporal signal‐to‐noise ratio (tSNR) of fMRI‐like 2D EPI from in‐vivo experiments with intentional movement (sequence parameters in Table 1). The tSNR maps were calculated before correction (Uncorrected), retrospectively corrected with CapTrack motion estimates (MoCo CapTrack), and retrospectively corrected with motion estimates from rigid body alignment (MoCo Rigid Align) using SPM12, Wellcome Centre UCL, UK. CapTrack per‐slice motion estimates were averaged to provide a single motion estimate for each volume. Both corrected series show improvements to tSNR over uncorrected images. Note that MoCo Rigid Align explicitly minimizes deviations across time and hence will be subject to effects of overfitting on the tSNR.
**Figure S2.** Retrospective motion correction of T1w 3D‐GRE on a structural phantom wearing the cap. Trial 3 (a‐f) compares retrospective correction using either CapTrack (c‐d) or interleaved navigators (e‐f). Trial 1 (g‐h) shows reference image quality without motion, with interleaved navigators. Trials with interleaved navigators (a‐h) were all affected by unwanted coherence between navigator and target sequence, due to comparatively long relaxation times of the phantom (T_1_/T_2_ ≈ 3000/170 ms). Coherence caused image artifacts in the target sequence (superimposed phase‐rolls) and less than desirable navigator performance (e‐f). Trial 4 (i‐l) demonstrates retrospective correction using CapTrack on images with motion. Trial 2 (m‐p) demonstrates retrospective correction using CapTrack on images without motion. A minor decrease in average edge strength (AES) is observed when applying CapTrack to a still phantom (o‐p), albeit without discernible decrease in visual sharpness
