# Supplementary material for: Tracking of rigid head motion during MRI using an EEG system
Source: Magn Reson Med. 2022 Apr 25;88(2):986–1001. doi: 10.1002/mrm.29251 (PMC9325421; doi:10.1002/mrm.29251)
Supplement: Supplementary file 1 — Figure S1. Map of temporal signal‐to‐noise ratio (tSNR) of fMRI‐like 2D EPI from in‐vivo experiments with intentional movement (sequence parameters in Table 1). The tSNR maps were calculated before correction (Uncorrected), retrospectively corrected with CapTrack motion estimates (MoCo CapTrack), and retrospectively corrected with motion estimates from rigid body alignment (MoCo Rigid Align) using SPM12, Wellcome Centre UCL, UK. CapTrack per‐slice motion estimates were averaged to provide a single motion estimate for each volume. Both corrected series show improvements to tSNR over uncorrected images. Note that MoCo Rigid Align explicitly minimizes deviations across time and hence will be subject to effects of overfitting on the tSNR. Figure S2. Retrospective motion correction of T1w 3D‐GRE on a structural phantom wearing the cap. Trial 3 (a‐f) compares retrospective correction using either CapTrack (c‐d) or interleaved navigators (e‐f). Trial 1 (g‐h) shows reference image quality without motion, with interleaved navigators. Trials with interleaved navigators (a‐h) were all affected by unwanted coherence between navigator and target sequence, due to comparatively long relaxation times of the phantom (T1/T2 ≈ 3000/170 ms). Coherence caused image artifacts in the target sequence (superimposed phase‐rolls) and less than desirable navigator performance (e‐f). Trial 4 (i‐l) demonstrates retrospective correction using CapTrack on images with motion. Trial 2 (m‐p) demonstrates retrospective correction using CapTrack on images without motion. A minor decrease in average edge strength (AES) is observed when applying CapTrack to a still phantom (o‐p), albeit without discernible decrease in visual sharpness [file MRM-88-986-s001.pdf]

1. Supplementary Material A

In practice, correlated translation and rotation parameters often occur because the center of rotations rarely coincides with the origin for motion parameters from standard realignment, which can be problematic in determining loop orientation dependency ($\boldsymbol{A}$) in eq. 6. In addition, rotations are non-linearly related to the elements in the affine transformation matrix. By decorrelating the movement parameters and thereby identifying the principal modes of movement prior to determining $\boldsymbol{A}$, the conditioning of the inversion can be improved by replacing eq. 6 with:

$$\boldsymbol{A} = [\Delta\boldsymbol{w}_{\boldsymbol{1}}, \Delta\boldsymbol{w}_{\boldsymbol{2}}, \dots, \Delta\boldsymbol{w}_{\boldsymbol{n}}]\boldsymbol{V}\boldsymbol{\Sigma}^{-1} \quad [1]$$

$$\boldsymbol{U}\boldsymbol{\Sigma}\boldsymbol{V}^{T} = [\mathcal{P}(\Delta\boldsymbol{r}_{\boldsymbol{1}}), \mathcal{P}(\Delta\boldsymbol{r}_{\boldsymbol{2}}), \dots, \mathcal{P}(\Delta\boldsymbol{r}_{\boldsymbol{n}})] \quad [2]$$

$\boldsymbol{U}, \boldsymbol{\Sigma}, \boldsymbol{V}$ is the singular value decomposition of $[\mathcal{P}(\Delta\boldsymbol{r}_{\boldsymbol{1}}), \mathcal{P}(\Delta\boldsymbol{r}_{\boldsymbol{2}}), \dots, \mathcal{P}(\Delta\boldsymbol{r}_{\boldsymbol{n}})]$, for n calibration positions. The operator $\mathcal{P}$ computes the affine 4×4 transformation matrix from $\Delta\boldsymbol{r}$ and vectorizes it.

After estimating $\boldsymbol{A}$ and $\dot{\tilde{\boldsymbol{G}}}(t)$, motion estimates based solely on loop recordings are estimated for a new set of weights, $\Delta\boldsymbol{w}$:

$$\Delta\boldsymbol{r} = \mathcal{P}^{-1}(\boldsymbol{U}\,\boldsymbol{A}^{+}\Delta\boldsymbol{w}) \quad [3]$$

$(\sim)^{+}$ indicates the Moore–Penrose (pseudo) inverse of a matrix. The non-linear operator $\mathcal{P}^{-1}$ transforms back into Cartesian coordinates and Euler angles ($\Delta\boldsymbol{r} = [\Delta x, \Delta y, \Delta z, \Delta\theta, \Delta\phi, \Delta\psi]$).

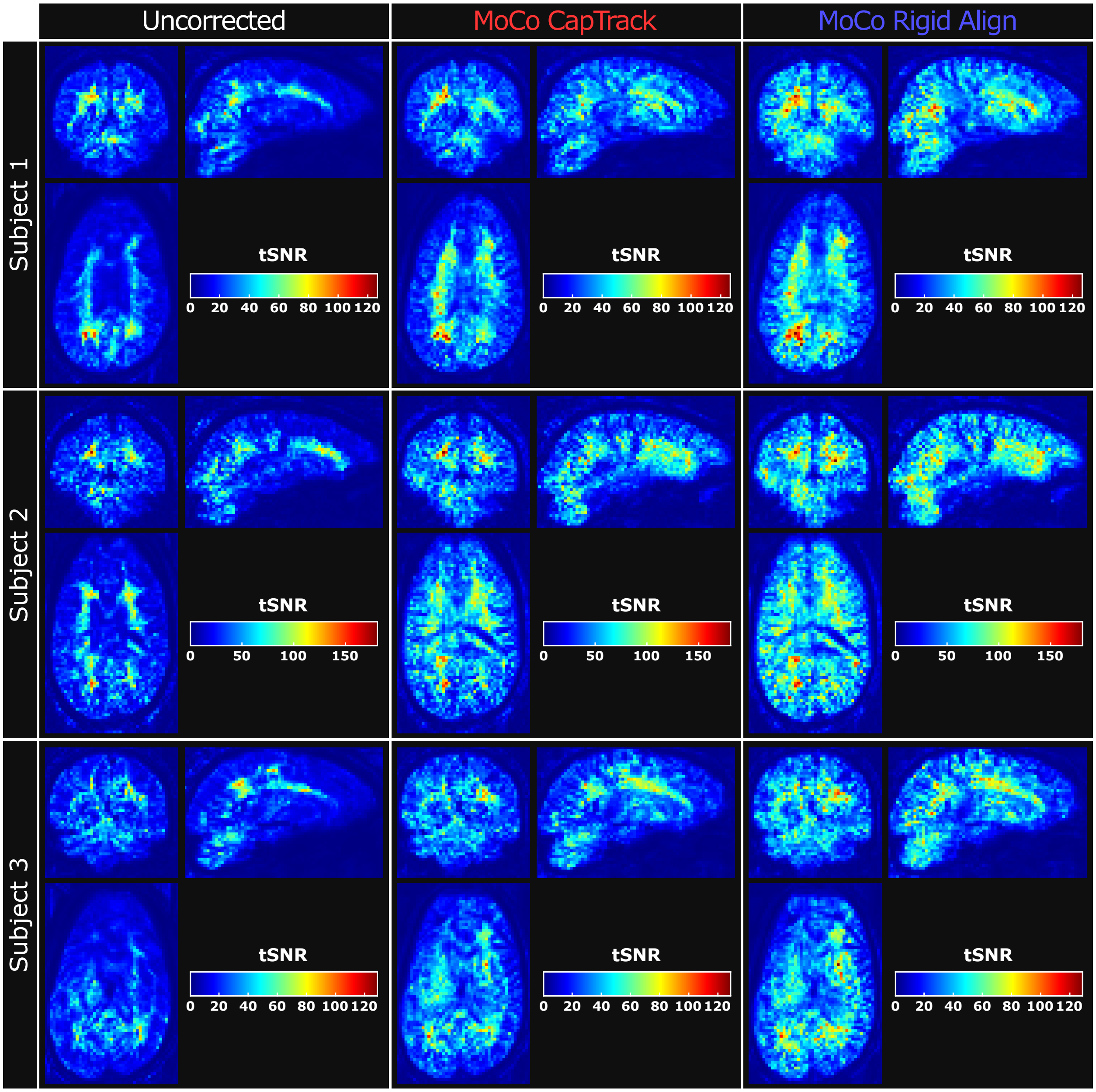

Supporting Information Figure S1: Map of temporal signal-to-noise ratio (tSNR) of fMRI-like 2D EPI from in-vivo experiments with intentional movement (sequence parameters in Table 1). The tSNR maps were calculated before correction (Uncorrected), retrospectively corrected with CapTrack motion estimates (MoCo CapTrack), and retrospectively corrected with motion estimates from rigid body alignment (MoCo Rigid Align) using SPM12, Wellcome Centre UCL, UK. CapTrack per-slice motion estimates were averaged to provide a single motion estimate for each volume. Both corrected series show improvements to tSNR over uncorrected images. Note that MoCo Rigid Align explicitly minimizes deviations across time and hence will be subject to effects of overfitting on the tSNR.

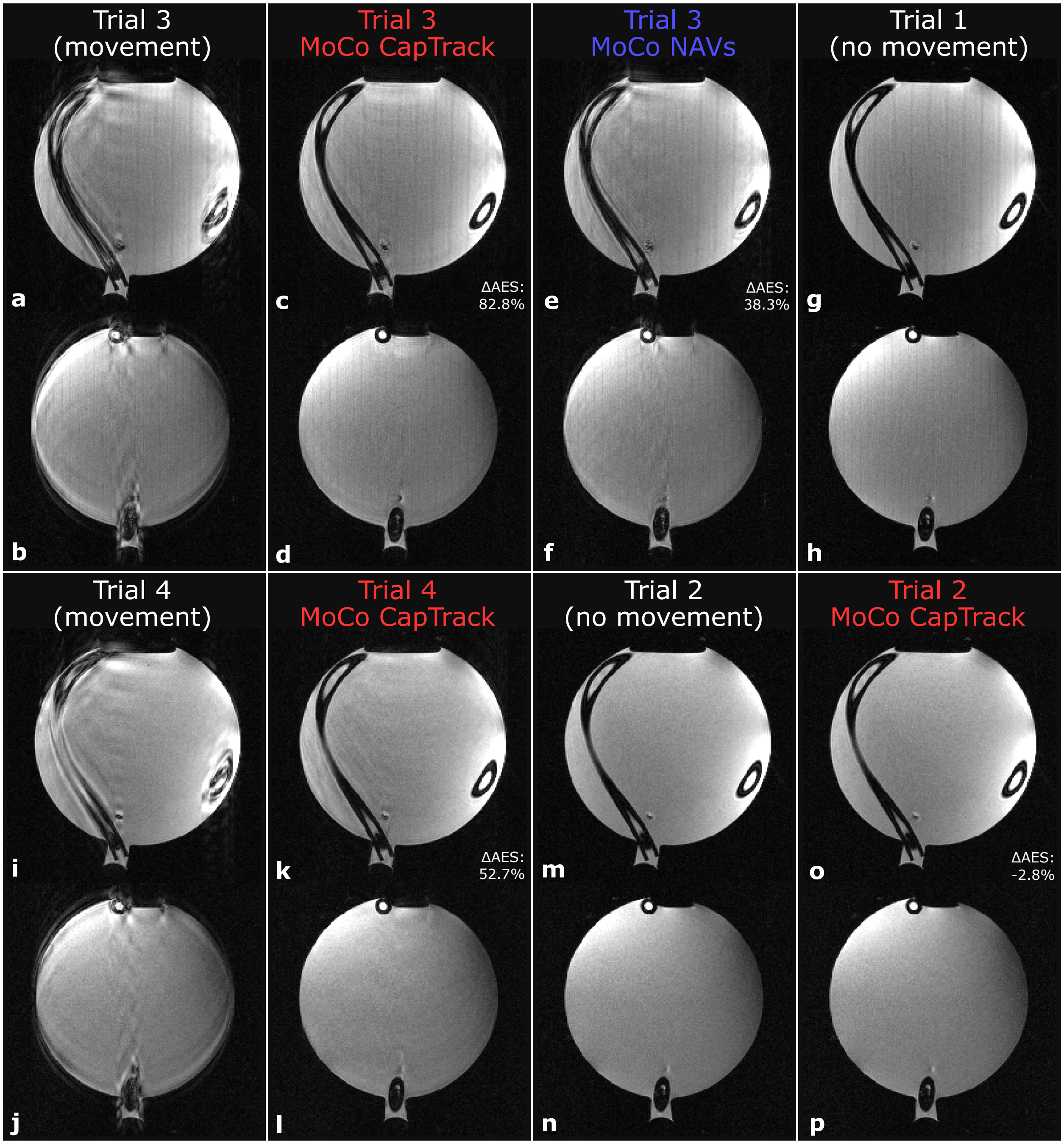

Supporting Information Figure S2: Retrospective motion correction of T1w 3D-GRE on a structural phantom wearing the cap. Trial 3 (a-f) compares retrospective correction using either CapTrack (c-d) or interleaved navigators (e-f). Trial 1 (g-h) shows reference image quality without motion, with interleaved navigators. Trials with interleaved navigators (a-h) were all affected by unwanted coherence between navigator and target sequence, due to comparatively long relaxation times of the phantom (T1/T2 ≈ 3000/170 ms). Coherence caused image artifacts in the target sequence (superimposed phase-rolls) and less than desirable navigator performance (e-f). Trial 4 (i-l) demonstrates retrospective correction using CapTrack on images with motion. Trial 2 (m-p) demonstrates retrospective correction using CapTrack on images without motion. A minor decrease in average edge strength (AES) is observed when applying CapTrack to a still phantom (o-p), albeit without discernible decrease in visual sharpness.
